# Differential roles of alexithymia components in linking autistic traits to personal distress and empathy concern

**DOI:** 10.3389/fpsyg.2026.1778225

**Published:** 2026-06-18

**Authors:** Xia Wang, Na Li, Yali Wang, Chenyu Shangguan, Huiling Zhou

**Affiliations:** 1 School of Education, Hunan First Normal University, Changsha, China; 2 School of Marxist, Zhejiang University of Finance and Economics, Hangzhou, China; 3 College of Education Science and Technology, Nanjing University of Posts and Telecommunications, Nanjing, China; 4 School of Education, Shanghai Normal University, Shanghai, China

**Keywords:** autistic traits, alexithymia components, personal distress, empathy concern, mediating effect

## Abstract

**Purpose:**

The alexithymia hypothesis proposes that the empathy deficits commonly found in ASD are driven mainly by coexisting alexithymia, not by ASD itself. Yet, empirical research has produced mixed evidence for this explanation. To further clarify this issue, the present study focused on how different component of alexithymia, such as, difficulty identifying feelings, difficulty describing feelings, and externally oriented thinking link autistic traits to two aspects of emotional empathy: personal distress and empathy concern.

**Method:**

A total of 730 participants took part in the investigation and completed three questionnaires assessing autistic traits, alexithymia, and emotional empathy.

**Result:**

Analyses revealed a divergent pattern in emotional empathy. Elevated autistic traits predicted heightened personal distress alongside diminished empathy concern. Mediation analyses further indicated that specific components of alexithymia accounted for these associations. Difficulty identifying feelings had a significant indirect effect on the relationship between autistic traits to heightened personal distress, whereas externally oriented thinking had a significant indirect effect on the relationship between autistic traits and empathy concern.

**Conclusion:**

These findings highlight the complex and multifaceted influence of alexithymia on empathy in individuals with elevated autistic traits and point to potential intervention strategies for enhancing empathy and social functioning in individuals on the autism spectrum.

## 1 Introduction

Autistic traits, deficits in social communication and interaction alongside restricted and repetitive patterns of behavior are not exclusive to Autism Spectrum Disorder (ASD), but are instead continuously distributed across the general population (De Groot and Van Strien, 2017; Sucksmith et al., 2011). Individuals with elevated autistic traits, even in non-clinical populations, are vulnerable to a range of mental, physical, and social functioning challenges, including increased psychological distress (Camus et al., 2023), heightened suicide risk (Pelton and Cassidy, 2017), poorer social skills (Jobe and White, 2007), and reduced prosocial behavior (Zhao et al., 2019). Non-clinical populations exhibiting elevated autistic traits show similar genetic, cognitive, and behavioral profiles to individuals with ASD (Gökçen et al., 2014; Kunihira et al., 2006; Lundström et al., 2012). Investigating autistic traits in the general population therefore offers a unique window into ASD mechanisms.

Individuals on the autism spectrum are commonly assumed to exhibit deficits in empathy, which are thought to play a key role in the social communication difficulties frequently observed within this population (Baron-Cohen, 2010). However, the specific manifestations and underlying mechanisms of empathy deficits remain unclear. This study examines the relationships between autistic traits and two aspects of emotional empathy, as well as the roles of different subcomponents of alexithymia in these associations. It aims to provide theoretical guidance for developing intervention strategies to enhance empathy and social functioning in individuals on the autism spectrum.

### 1.1 Autism and empathy

Empathy, defined as the ability of an individual to understand and vicariously experience the mental states of others, serves as a cornerstone of social interaction and interpersonal communication (Decety and Jackson, 2004). Empathy is generally divided into two main components: cognitive empathy and emotional empathy (Davis, 1983). The former refers to the capacity to recognize and infer others’ beliefs, intentions, and emotional states (Decety and Lamm, 2006), whereas the latter encompasses two aspects: personal distress (PD) and empathy concern (EC). PD denotes self-focused feelings of anxiety or discomfort triggered by another person’s suffering, while EC represents other-focused feelings of compassion and sympathy in response to someone else’s distress (Eisenberg and Eggum, 2009).

Researchers have extensively examined empathy among individuals exhibiting either clinical ASD or elevated autistic traits in generally populations. Consistently, they have reported impairments in cognitive empathy, whereas no consistent conclusions have been reached regarding emotional empathy (Fatima and Babu, 2023). Some studies report deficits (Liu et al., 2023; Mazza et al., 2014), whereas other studies report comparable or even elevated levels relative to typical groups (Zhang et al., 2023; Fan et al., 2014). A potential source of this discrepancy lies in how emotional empathy is conceptualized, with some investigations examine only EC (Dziobek et al., 2008; Trimmer et al., 2017) or PD (Fan et al., 2014; Santiesteban et al., 2021), and others combining them as a single construct (Mazza et al., 2014; Mul et al., 2018). In some studies, EC and PD has been examined separately and simultaneously, showing that, compared with typical individuals, those with ASD exhibit lower levels of other-oriented EC (Santiesteban et al., 2021) and higher levels of self-oriented PD (Dziobek et al., 2008). Comparable results have been observed in non-clinical samples, with autistic traits inversely related to EC but positively related to PD (Aaron et al., 2015).

### 1.2 The alexithymia hypothesis

ASD and alexithymia have a high comorbidity rate. In community samples, alexithymia rarely exceeds 5%, whereas its prevalence among individuals with ASD is estimated to be around 50% (Kinnaird et al., 2019). Alexithymia is a subclinical trait marked by difficulty identifying and describing emotions, alongside a tendency to think in an externally oriented manner (Bagby et al., 1994). According to the “shared-networks hypothesis” of empathy, perceiving others’ emotions engages brain regions similar to those activated during one’s own emotional experience, constituting the neural basis of empathy (Decety and Jackson, 2004; Singer et al., 2004). This suggests that difficulties in processing personal affect may impair one’s ability to accurately understand and respond to others’ emotions. Empirical research also reported a negative association between alexithymia and empathy (Banzhaf et al., 2018; Ferguson et al., 2023; Grynberg et al., 2010).

The alexithymia hypothesis proposes that the empathy deficits commonly found in ASD are driven mainly by coexisting alexithymia, not by ASD itself (Bird and Cook, 2013). Some studies have offered empirical evidence consistent with this perspective. For instance, research has shown that when alexithymia levels are controlled for, individuals with ASD exhibit empathy levels comparable to those of typical individuals (Mul et al., 2018; Raman et al., 2023; Butera et al., 2023). Other studies have shown that alexithymia serves as a mediator in the relationship between autistic traits and empathy (Aaron et al., 2015). However, not all findings align with this hypothesis. For instance, Shah et al. (2019) indicated that both autistic traits and alexithymia independently predict empathy, and that autistic traits even exert a stronger predicter on empathy than alexithymia does.

### 1.3 Distinct components of alexithymia

To date, the distinct features of alexithymia’s subcomponents have received limited attention in the literature. Alexithymia is multidimensional, encompassing three components: difficulty in identifying feelings (DIF), difficulty in describing feelings (DDF), and an externally oriented thinking style (EOT, Bagby et al., 1994). DIF involves difficulties in the recognition of internal emotions and their differentiation from the bodily sensations elicited by emotional arousal. DDF reflects difficulty in verbally expressing emotions. EOT refers to a thinking style in which attention is primarily directed toward external events instead of internal emotional experiences (Bagby et al., 1994). Although this three-factor structure has been validated empirically (Zhu et al., 2007), notable heterogeneity exists among the three components. For example, DIF and DDF are strongly associated with psychological and somatic health problems, whereas EOT shows weaker associations and could potentially confer a protective effect in some contexts (Bilge and Tankut, 2022; Declercq et al., 2010; Liu et al., 2024). Moreover, neuroimaging studies indicates that each component is underpinned by distinct neuroanatomical substrates (Goerlich-Dobre et al., 2015).

Distinct alexithymia components may play different roles in the associations between autistic traits and two aspects of emotional empathy. DIF and DDF reflect deficits in the ability to interpret emotional experiences as identifiable and verbally expressible emotional states (Bagby et al., 1994). According to the Self–Other Model of Empathy (SOME), empathy distress arises when the perceiver cannot explicitly tags their affective state as originating from the other person (Bird and Viding, 2014). Individuals with high DIF and DDF, due to impaired emotional awareness, may misattribute the origin of their emotions (self or other) in empathic contexts, thereby resulting in heightened self-focused arousal and increased susceptibility to PD. EOT is characterized by a predominant focus on external events (Bagby et al., 1994), whereas EC entails sincere concern for and interest in others’ internal states (Eisenberg and Eggum, 2009). Therefore, individuals with high EOT may tend to show reduced EC. Empirical evidence supports these distinctions. Using factor analysis, Grynberg et al. (2010) revealed shared variance between empathy and alexithymia. Specifically, DIF, DDF, and PD loaded on a common factor, whereas EOT and EC loaded on separate factors, suggesting two distinct pathways linking alexithymia to empathy. More recently, Di et al. (2025) employed network analysis to investigate the complex relationships among autistic traits, alexithymia, and empathy, and found strong positive associations between DIF and PD, as well as negative associations between EOT and EC.

### 1.4 This study

Although a growing body of research has examined the role of alexithymia in the relationship between autistic traits and empathy, existing findings remain inconsistent (Mul et al., 2018; Raman et al., 2023; Butera et al., 2023; Aaron et al., 2015; Shah et al., 2019). One possible reason for these inconsistent findings is that most previous studies have treated alexithymia as a unitary construct, potentially obscuring the distinct roles of its subcomponents. The subcomponents of alexithymia (DIF, DDF, EOT) may differentially relate to specific facets of empathy (Grynberg et al., 2010; Di et al., 2025). As a result, it remains unclear which components of alexithymia are primarily responsible for linking autistic traits to empathy outcomes such as PD and EC.

To address this gap, the present study examined the unique contributions of DIF, DDF, and EOT to the associations between autistic traits and PD/EC. By adopting a multidimensional perspective on alexithymia, this study provides a more fine-grained understanding of the emotional processing mechanisms underlying empathy deficits associated with autistic traits and may help explain inconsistencies in previous findings. We propose following hypotheses: (1) autistic traits are expected to be positively related to PD but negatively related to EC (H1); (2) DIF and DDF are hypothesized to mediate the link between autistic traits and PD (H2); (3) EOT is proposed to mediate the association between autistic traits and EC (H3).

## 2 Methods

### 2.1 Participants and procedures

A total of 730 undergraduate students (53.88% female) were recruited via cluster sampling from four universities in China, whose ages ranged from 16 to 26 years (*M* = 19.81, SD = 1.23). Participation was voluntary, either without compensation or as part of a course requirement. Seventeen participants were excluded due to extreme response times or uniform responses across items, leaving 713 participants for analysis. All participants reported no history of mental illness and no use of medication during the survey period.

Data were collected through the online survey platforma.^1^ After providing demographic information, participants completed three Chinese language questionnaires: the Autism Spectrum Quotient (AQ, Liu, 2008), the 20-item Toronto Alexithymia Scale (TAS-20, Zhu et al., 2007), and the personal distress and empathy concern subscales of the Interpersonal Reactivity Index (IRI, Rong et al., 2010). The order of questionnaire administration was randomized to control for order effects. The study protocol was approved by the Ethics Committee of Hunan First Normal University.

### 2.2 Measures

*Autism Trait* We employed the 50-item AQ (Baron-Cohen et al., 2001) to evaluate autistic traits. Each item was rated on a four-point format ranging from 1 (definitely agree) to 4 (definitely disagree), and scores were subsequently binarized following established scoring guidelines (agreement responses were assigned 1 and disagreement 0). A higher cumulative score was indicative of stronger autistic tendencies. The Chinese adaptation has been shown to perform adequately in psychometric testing (Liu, 2008), and in the current sample, internal consistency was acceptable (*α* = 0.65).

*Alexithymia* We evaluated alexithymia with the TAS-20 (Bagby et al., 1994). This instrument comprises three subdimensions (DIF, DDF, EOT), consisting of 7, 5, and 8 items, respectively. Each item is rated on a 5-point format ranging from 1 (strongly disagree) to 5 (strongly agree). Higher composite scores reflect greater alexithymic tendencies. The Chinese adaptation has been shown to perform adequately in psychometric testing (Zhu et al., 2007), and internal consistency in the present study was satisfactory (α = 0.82).

*Personal Distress and Empathy Concern* We use the IRI (Davis, 1983) to assessed personal distress and empathy concern. The Chinese adaptation has been shown to perform adequately in psychometric testing (Rong et al., 2010). In this study, two subscales were administered: PD (seven items) and EC (seven items). All items were rated on a 5-point scale ranging from 1 (does not describe me well) to 5 (describes me very well), with higher scores indicating greater levels of the respective construct. Cronbach’s alpha coefficients in the present sample were 0.75 for PD and 0.78 for EC.

### 2.3 Statistical analysis

Data analyses were performed with IBM SPSS Statistics 27.0. Procedures included checking data normality and multicollinearity, testing for common method variance, and conducting descriptive as well as Pearson correlation analyses. To examine the proposed mediation effects, two path analyses were conducted using the PROCESS macro (Model 4) for SPSS with the dependent variable differing across models (PD and EC respectively, Hayes, 2017). In both models, autistic traits were served as the independent variable, while DIF, DDF, and EOT were entered as parallel mediators to assess their unique contributions. Gender and age were controlled. The indirect effects were estimated using a bootstrap resampling procedure with 10,000 iterations. Given the implementation of two mediation models, a Bonferroni adjustment was employed to control increased family-wise error rate. Accordingly, the adjusted threshold for statistical significance was set at *p* < 0.025 (i.e., 0.05 divided by 2) and 97.5% CIs were used in the mediation analyses. When the confidence interval encompassed zero, the indirect effect was considered non-significant.

In addition, AQ comprises five subdimensions: social skills (SS), communication (CM), attention switching (AS), attention to detail (AD), and imagination (IM). Given that previous studies have suggested that different aspects of autistic traits may show distinct associations with alexithymia and empathy (Di et al., 2025), the present study further conducted supplementary analyses to examine whether the relationships among autistic traits, alexithymia, and empathy were consistent across different AQ subdimensions.

## 3 Results

### 3.1 Preliminary analyses

Normality was assessed via skewness and kurtosis, and all values fell within the recommended thresholds of |2| and |7|, respectively (Byrne, 2010), indicating that the distribution of all study variables met the assumption of normality. Multicollinearity among the independent variable and the three mediators was examined. The variance inflation factors (VIFs) for all predictors were below the conservative cutoff of 3.0 (O’Brien, 2007), indicating no multicollinearity-related bias in the regression estimates. Harman’s one-factor test was performed to examine the possibility of common method variance. Results showed that the first factor explained merely 9.40% of the overall variance, far below the commonly accepted 40% benchmark (Podsakoff et al., 2003). These findings imply that common method bias did not pose a significant effect in the present study.

### 3.2 Descriptive statistics and correlation analysis

Table 1 displays the means, standard deviations, and intercorrelations for all study variables. The results show a positive association between autistic traits and both the total alexithymia score and its three components. Autistic traits were positively correlated with PD but negatively correlated with EC. The total alexithymia score and DDF and EOT subcomponents were negatively correlated with EC. The total alexithymia score and all components were positively correlated with PD.

**Table 1:** Means, standard deviations, and correlations among variables in this study (*N* = 713).

Variables	*M*	SD	1	2	3	4	5	6	7
1. Autistic traits	22.62	5.57	1						
2. Alexithymia	55.33	9.02	0.41**	1					
3. DIF	19.76	5.14	0.37**	0.89**	1				
4. DDF	14.68	2.98	0.40**	0.83**	0.69**	1			
5. EOT	20.87	3.30	0.18**	0.60**	0.25**	0.26**	1		
6. EC	25.95	3.96	−0.13**	−0.10**	−0.02	−0.09*	−0.15**	1	
7. PD	24.32	3.60	0.28**	0.34**	0.36**	0.28**	0.11**	0.30**	1

*p < 0.05, **p < 0.01.

### 3.3 Mediation analysis

#### 3.3.1 Personal distress

**Figure 1:**
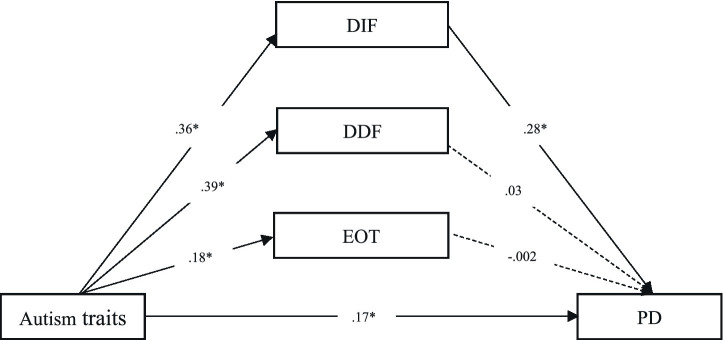
Mediation model predicting PD, with gender and age included as covariates. *p < 0.025. Solid lines indicate significant effects, and dashed lines indicates non-significant effects.

The first mediation analysis examined how the three components of alexithymia contributed to the higher levels of PD linked to autistic traits. The results are presented in Table 2 and Figure 1. More prominent autism traits were significantly predicted heightened DIF, DDF, EOT and PD. Heightened DIF significantly predicted elevated PD, whereas DDF and EOT were not significantly predicted elevated PD.

**Table 2:** Standardized regression beta estimates, standard errors, *p* values, and 97.5% confidence intervals for the mediation model predicting PD.

Regression paths	*β*	SE	*p* value	Confidence intervals	
				Lower bound	Upper bound
Autism traits → DIF	0.36*	0.03	<0.001	0.26	0.41
Autism traits → DDF	0.39*	0.02	<0.001	0.17	0.25
Autism traits → EOT	0.18*	0.02	<0.001	0.06	0.16
DIF → PD	0.28*	0.03	<0.001	0.12	0.27
DDF → PD	0.03	0.06	0.47	−0.09	0.17
EOT → PD	−0.002	0.04	0.96	−0.09	0.08
Autism traits → PD	0.17*	0.02	<0.001	0.06	0.16

*β*, Standardized beta estimates; SE, standard errors, *p < 0.025.

Analysis of the indirect effect showed that the DIF served as a significant mediator between autistic traits and PD [*β* = 0.10, *SE* = 0.02, 97.5% CI = (0.05, 0.15)]. The DDF [*β* = 0.01, *SE* = 0.02, 97.5%CI = (−0.04, 0.06)] and EOT [*β* = −0.0003, *SE* = 0.007, 97.5%CI = (−0.02, 0.02)] did not significantly mediated this relationship. In addition, the total effect [*β* = 0.28, *SE* = 0.02, 97.5%CI = (0.13, 0.23)] and direct effect [*β* = 0.17, *SE* = 0.02, 97.5%CI = (0.06, 0.16)] of autistic traits on PD were significant.

#### 3.3.2 Empathy concern

**Figure 2:**
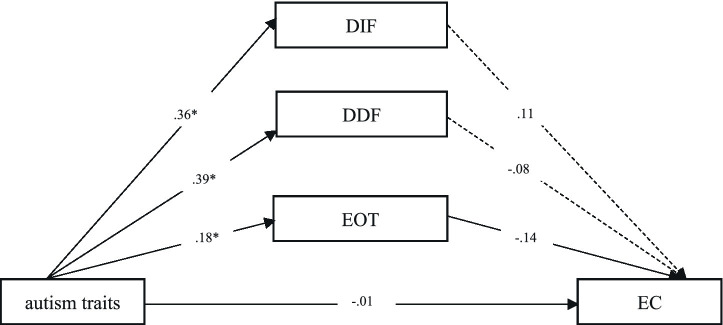
Mediation model predicting EC, with gender and age included as covariates. *p < 0.025. Solid lines indicate significant effects, and dashed lines indicates non-significant effects.

The second mediation analysis examined how the three components of alexithymia contributed to the reduced EC linked to autistic traits. The findings are presented in Table 3 and Figure 2. More prominent autism traits were significantly predicted heightened DIF, DDF, EOT and reduced EC. Heightened EOT significantly predicted reduced EC, whereas DIF and DDF were not significantly predicted EC.

**Table 3:** Standardized regression beta estimates, standard errors, *p* values, and 97.5% confidence intervals for mediation model predicting EC.

Regression paths	*β*	SE	*p* value	Confidence Intervals	
				Lower bound	Upper bound
Autism traits → DIF	0.36*	0.03	<0.001	0.26	0.41
Autism traits → DDF	0.39*	0.02	<0.001	0.17	0.25
Autism traits → EOT	0.18*	0.02	<0.001	0.06	0.16
DIF → EC	0.11	0.04	0.04	−0.007	0.17
DDF → EC	−0.08	0.07	0.12	−0.27	0.05
EOT → EC	−0.14*	0.05	<0.001	−0.27	−0.06
Autism traits → EC	−0.10*	0.03	0.013	−0.14	−0.007

*β*, Standardized beta estimates; SE, standard errors, *p < 0.025.

Analysis of the indirect effect showed that the EOT served as a significant mediator between autistic traits and EC [*β* = −0.02, *SE* = 0.01, 97.5%CI = (−0.05, −0.005)]. The DIF [*β* = 0.04, *SE* = 0.02, 97.5%CI = (−0.006, 0.09)] and DDF [*β* = −0.03, *SE* = 0.02, 97.5%CI = (−0.08, 0.02)] did not significantly mediated this relationship. In addition, the total effect [*β* = −0.11, *SE* = 0.03, 97.5%CI = (−0.14, −0.03)] and direct effect [*β* = −0.10, *SE* = 0.03, 97.5%CI = (−0.14, −0.007)] of autistic traits on EC were significant.

### 3.4 Supplementary analyses results

Supplementary analyses using AQ subdimensions yielded a pattern of results that was largely consistent with those obtained using the total AQ score. Specifically, for the SS, AS, CM, and IM dimensions, DIF served as a significant mediator between autistic traits and PD. At the same times, EOT served as a significant mediator between autistic traits and EC. An exception was observed for the AD dimension. For AD, the indirect effect on PD via DIF was not significant, whereas the indirect effect on EC via EOT was significant. Notably, AD positively predicted EC through EOT, which was opposite in direction to the indirect effect observed for the total AQ score. Detailed results are presented in Supplementary materials.

## 4 Discussion

This study investigated the associations between autistic traits and two aspects of emotional empathy (PD and EC), as well as the roles of alexithymia components (DIF, DDF, and EOT) in these relationships. Consistent with our hypotheses, the results showed that autistic traits positively predicted PD and negatively predicted EC (support H1). Moreover, DIF mediate the association between autistic traits and PD (partly support H2) and EOT mediate the association between autistic traits and EC (support H3).

### 4.1 Opposite associations of autistic traits with personal distress and empathy concern

This study simultaneously examined two aspects of emotional empathy: PD and EC, and found that autistic traits were positively associated with PD but negatively associated with EC. These results align with prior studies (Aaron et al., 2015; Dziobek et al., 2008; Santiesteban et al., 2021). PD and EC represent two typical emotional responses when witnessing others’ suffering (Eisenberg and Eggum, 2009). PD, though a measure of emotional empathy, has been linked to maladaptive outcomes like rumination and internalizing symptoms (Joireman, 2004; Tone and Tully, 2014). In empathic situations, individuals with high levels of PD may withdraw from others’ suffering in order to regulate their own emotional overload, thereby limiting empathic engagement and prosocial responses (Kim and Han, 2018). In contrast, EC is associated with more adaptive traits such as extraversion and agreeableness (Song and Shi, 2017) and is known to promote costly prosocial behaviors (FeldmanHall et al., 2015). Findings from the current investigation suggest that those with higher autistic traits exhibit deficit in emotional empathy, reflected in elevated levels of negatively self-oriented PD and reduced levels of positively other-oriented EC. These results offer a potential explanation for the inconsistencies observed in previous research (Zhang et al., 2023; Fan et al., 2014; Liu et al., 2023; Mazza et al., 2014) and highlight the need for clearer conceptualization and operationalization of emotional empathy in researches.

### 4.2 Differential role of alexithymia components between autistic traits and two aspects of emotional empathy

This study takes an initial effort to explore how alexithymia subcomponents contributes to the associations between autistic traits and two aspects of emotional empathy. Our results indicated that autistic traits were positively linked to alexithymia and its individual dimensions. These findings are consistent with prior research, which shown elevated levels of overall alexithymia (Griffin et al., 2016) and its subcomponents in ASD (Ferguson et al., 2023). In addition, alexithymia displayed an opposite association pattern with PD and EC, being positively associated with PD across all subcomponents, but negatively associated with EC, except for DIF. This outcome also mirrors findings reported in earlier research. For instance, Butera et al. (2023) were the first to distinguish between PD and EC in their analysis, showing that alexithymia was positively linked to PD and inversely related to EC in those with ASD.

Furthermore, our findings support the mediating role of DIF in the relationship between autistic traits and PD, suggesting that difficulties in emotional awareness may be related to heightened self-oriented emotional distress among individuals with elevated autistic traits. We also found EOT mediate the relationship between autistic traits and EC, suggesting that a cognitive style characterized by reduced attention to internal emotional states may be linked to lower levels of other-oriented empathic responses among individuals with elevated autistic traits.

These findings can be understood from the perspective of the SOME (Bird and Viding, 2014). According to this model, effective empathic responding requires individuals to establish emotional representations by understanding others’ situations or recognizing their emotional states. Individuals high in EOT tend to focus on external and concrete information while paying limited attention to emotional experiences (Bagby et al., 1994). Such reduced emotional engagement may impair the processing of others’ affective cues, thereby weakening the generation of other-oriented empathic concern.

The SOME model also proposes that empathy requires a clear distinction between self- and other-related emotional representations (Bird and Viding, 2014). Individuals high in DIF may form vague or poorly differentiated representations of their own emotional states (Bagby et al., 1994). Consequently, when confronted with others’ distress, they may experience difficulty distinguishing whether the elicited emotions originate from themselves or from others. This reduced self-other differentiation may increase susceptibility to emotional contagion, ultimately leading to heightened self-focused personal distress. In addition, from the perspective of emotion regulation frameworks, the ability to identify and label emotions is a prerequisite for adaptive regulation strategies such as cognitive reappraisal (Gross, 2015). When emotional states are poorly identified, individuals may be less able to modulate their emotional responses, leading to sustained or amplified arousal in response to others’ suffering. This combination of impaired self-other distinction and reduced regulatory capacity may explain why DIF is particularly associated with increased PD.

These findings support the alexithymia hypothesis by suggesting that deficits in emotional processing may underlie atypical empathy among individuals with elevated autistic traits (Bird and Cook, 2013). More importantly, our findings further refine this hypothesis by suggesting that different emotional processing impairments may underlie distinct empathic difficulties associated with autistic traits.

However, no mediating effect of DDF was observed in the link between autistic traits and PD. This may be because DDF primarily involves difficulties in verbalizing emotions, which primarily affect social communication rather than emotional awareness in a way that link to personal distress (Koppelberg et al., 2023).

### 4.3 Distinct patterns across dimensions of autistic traits

The supplementary analyses conducted at the level of AQ subdimensions indicated that the SS, AS, CM, and IM dimensions showed result patterns that were generally consistent with those obtained using the total AQ score. However, AD showed a markedly different result patterns. Specifically, the indirect association between AD and PD via DIF was not significant, whereas the indirect association between AD and EC via EOT was significant, but in the opposite direction to that observed for the total AQ score.

Factor-analytic studies have suggested that the AQ consists of two broader factors: SS, AS, CM, and IM primarily load onto a social factor, whereas AD forms a relatively independent factor (Hoekstra et al., 2008). Studies have shown that the social components of autistic traits have been associated with increased PD, whereas the non-social components appear to be unrelated to PD (Svedholm-Häkkinen et al., 2018). Similarly, studies examining the relationship between autistic traits and alexithymia have found that the social components are positively associated with alexithymia, whereas the non-social components show little or no association with alexithymia (Liss et al., 2008). Our findings are consistent with these previous studies, further suggesting that the social components (e.g., SS, AS, CM, and IM) of autistic traits may be more strongly related to alexithymia-related emotional difficulties and heightened personal distress.

Notably, a different pattern emerged for the association of AD and EC. One possible explanation is that AD reflects a cognitive style characterized by enhanced sensitivity to subtle information and increased attentional engagement, rather than social–emotional impairment (Smith and Milne, 2009). Previous studies have indicated individuals high in AD tend to exhibit enhanced perceptual sensitivity, superior detection of fine-grained environmental changes (Baron-Cohen et al., 2009; Stevenson et al., 2017). Therefore, individuals high in AD may be more likely to notice subtle affective cues of others in empathic situation, which could facilitate emotional processing and reduce externally oriented thinking, thereby promoting EC.

### 4.4 Implications

The present study offers important theoretical and practical implications. Our findings provide support for the alexithymia hypothesis (Bird and Cook, 2013), showing that alexithymia significantly mediates the relationship between autistic traits and emotional empathy, highlighting its role as a key mechanism underlying empathy deficits in individuals with higher autistic traits. Importantly, the persistent direct effect of autistic traits on emotional empathy indicates that additional mechanisms beyond alexithymia also contribute. Moreover, examining alexithymia at the subcomponent level reveals that different facets play distinct mediating roles across the two dimensions of emotional empathy. Together, these results underscore the need for a more nuanced framework that captures the complex and multifaceted influence of alexithymia on empathy in individuals with elevated autistic traits. Such an approach may help clarify the inconsistent evidence observed in existing research (Aaron et al., 2015; Shah et al., 2019).

Practically, the findings provide specific guidance for developing empathy interventions tailored to individuals with elevated autistic traits. For those exhibiting intense self-focused emotional responses in empathic situations, intervention efforts may benefit from targeting the enhancement of emotional awareness and emotional recognition. Differently, for individuals who display low levels of empathy concern or prosocial responsiveness, interventions may focus on strengthening introspective abilities and fostering deeper engagement with internal emotional experiences.

### 4.5 Limitation and further direction

Several limitations warrant consideration in this study. First, this study examined autistic traits in a non-clinical sample, leaving the generalizability to individuals with a formal ASD diagnosis uncertain. Future research should verify whether the same patterns emerge in clinical populations. Second, the cross-sectional design limits causal interpretation (Maxwell et al., 2011). Despite the theoretical and empirical support for our mediation model, longitudinal or experimental research is necessary to confirm causality. Third, all data were collected using self-report measures, which may be vulnerable to social desirability bias and limitations in introspective accuracy. The latter may be particularly relevant for constructs such as alexithymia and empathy, as difficulties in identifying and describing emotions may affect participants’ ability to accurately report their own emotional experiences. Future research should adopt multimethod approaches, such as incorporating observational, physiological, and informant-report measures, to provide more comprehensive and robust assessments. Fourth, the internal consistency of the AQ total score in the present sample was relatively modest. Lower reliability may increase measurement error and attenuate the observed associations among variables (Fan, 2003), suggesting that the findings should be interpreted with caution. Nevertheless, the overall pattern of results was theoretically meaningful and generally consistent with previous literature. In addition, the supplementary analyses at the level of AQ subdimensions yielded largely similar patterns of results, providing some support for the robustness of the findings. Future research should employ measures with stronger psychometric properties to further validate and replicate the present findings.

## 5 Conclusion

This study offers the empirical support that autistic traits are differentially associated with two aspects of emotional empathy, increasing PD while reducing EC. Crucially, these associations are mediated by distinct components of alexithymia: DIF links autistic traits to greater PD, whereas EOT links autistic traits to reduced EC. By dissecting alexithymia into its subcomponents, this study offers novel and fine-grained insights into the mechanisms underlying empathy deficits in individuals with elevated autistic traits, highlighting alexithymia as a multidimensional explanatory factor and a promising target for intervention.

## Data Availability

The raw data supporting the conclusions of this article will be made available by the authors, without undue reservation.

## Footnotes

1
https://www.wjx.cn/
