# Supplementary material for: Differential roles of alexithymia components in linking autistic traits to personal distress and empathy concern
Source: Front Psychol. 2026 Jun 18;17:1778225. doi: 10.3389/fpsyg.2026.1778225 (PMC13323232; doi:10.3389/fpsyg.2026.1778225)
Supplement: Supplementary file 1 [file Presentation_1.PDF]

Supplementary Materials

Supplementary analyses were conducted to further examine the relationships between AQ subdimensions, alexithymia subdimensions, and emotional empathy. These analyses included both correlation and mediation analyses. Overall, the findings were largely consistent with those based on the AQ total score. However, attention to detail demonstrated a distinct pattern, showing no significant indirect effect on PD via DIF, but a positive indirect effect on EC via EOT. The results of the correlation analyses are presented in Table S1, and the results of the mediation analyses are shown in Figures S1–10.

Results of Correlation analysis

Table S1 Correlations between autistic traits subdimensions, alexithymia subdimensions, and emotional empathy.

Variables	1	2	3	4	5	6	7	8	9	10
1.SS	1									
2.AS	.35**	1								
3.AD	-.20*	-.03	1							
4.CM	.52**	.28**	-.20**	1						
5.IM	.23**	.08*	-.08*	.22**	1					
6.DIF	.21**	.26**	.04	.34**	.13**	1				

7.DDF	.29**	.22**	-.07	.43**	.20**	.69**	1			
8.EOT	.15**	.12**	-.20**	.26**	.17**	.25**	.26**	1		
9.EC	-.14**	-.006	.12**	-.16**	-.15**	-.02	-.09*	-.15**	1	
10.PD	.20**	.38**	-.11**	.25**	.01	.36**	.28**	.11**	.30**	1

Note: SS = Social Skills; AS = Attention Switching; AD = Attention to Detail; CM = Communication; IM = Imagination; DIF = Difficulty Identifying Feelings; DDF = Difficulty Describing Feelings; EOT = Externally Oriented Thinking; EC = Empathic Concern; PD = Personal Distress. *p < .05, ** p < .01.

Results of the mediation analysis with SS as the independent variable, DIF, DDF, and EOT as mediators, and PD as the dependent variable.

The mediation path coefficient diagram is presented in Figure S1. More prominent SS were significantly predicted heightened DIF (β = .21, SE = .07, CI = [.28, .57]), DDF(β = .29, SE = .04, CI = [.25, .41]) and EOT(β = .15, SE = .05, CI = [.09, .28]). Heightened DIF significantly predicted elevated PD (β = .32, SE = .03, CI = [.16, .29]), whereas DDF (β = .01, SE = .06, CI = [-.10, .13]) and EOT(β = .01, SE = .04, CI = [-.06, .09]) were not significantly predicted elevated PD. The indirect effect showed that the DIF served as a significant mediator between SS and PD (β = .07, SE = .02, CI = [.04, .10]). The DDF and EOT did not significantly mediated this relationship.

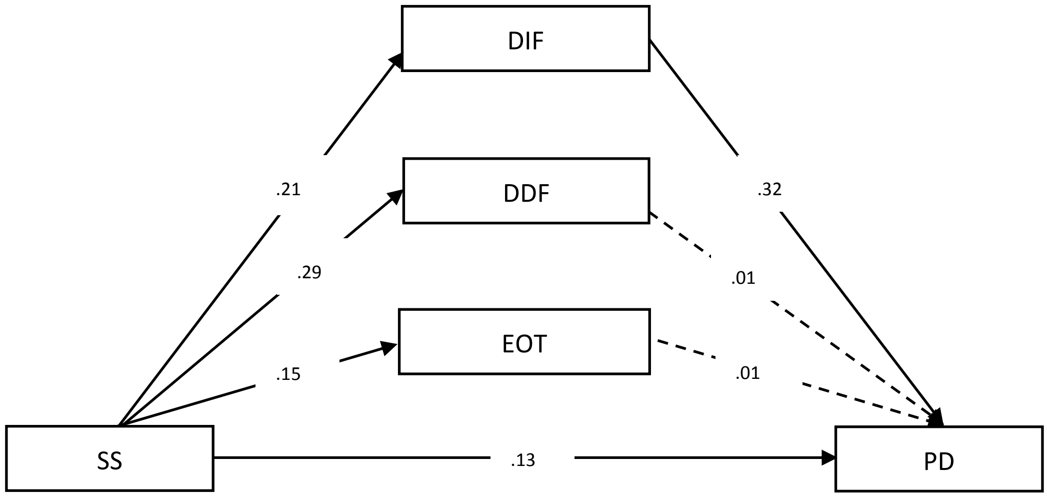

Figure S1 Mediation path coefficients with SS as the independent variable, DIF, DDF, and EOT as mediators, and PD as the dependent variable.

Results of the mediation analysis with AS as the independent variable, DIF, DDF, and EOT as mediators, and PD as the dependent variable.

The mediation path coefficient diagram is presented in Figure S2. More prominent AS were significantly predicted heightened DIF (β = .26, SE = .10, CI = [.54, .94]), DDF(β = .22, SE = .06, CI = [.25, .48]) and EOT(β = .12, SE = .07, CI = [.09, .35]). Heightened DIF significantly predicted elevated PD (β = .26, SE = .03, CI = [.47, .74]), whereas DDF (β = .03, SE = .06, CI = [-.08, .14]) and EOT(β = .005, SE = .04, CI = [-.07, .08]) were not significantly predicted elevated PD. The indirect effect showed that the DIF served as a significant mediator between AS and PD (β = .07, SE = .02, CI = [.04, .10]). The DDF and EOT did not significantly mediated this relationship.

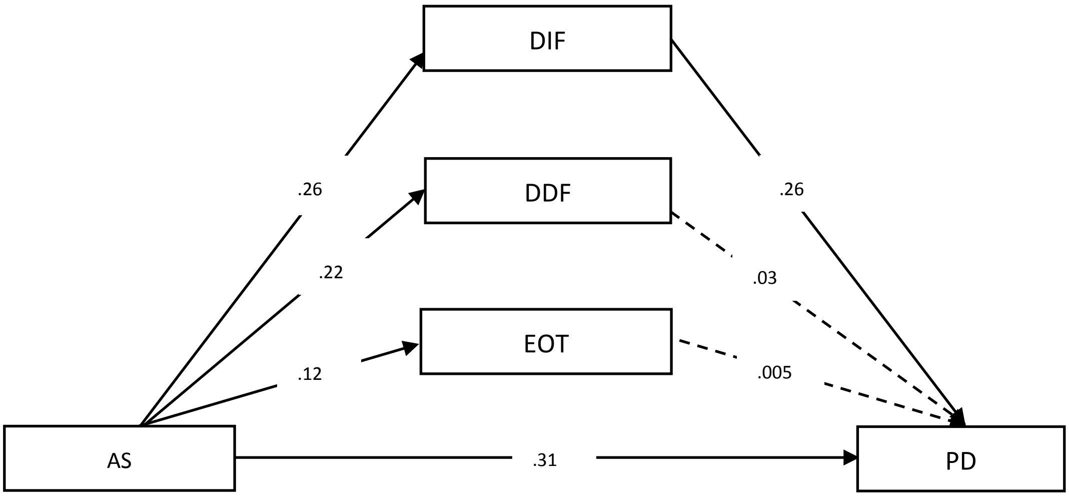

Figure S2 Mediation path coefficients with AS as the independent variable, DIF, DDF, and EOT as mediators, and PD as the dependent variable.

Results of the mediation analysis with CM as the independent variable, DIF, DDF, and EOT as mediators, and PD as the dependent variable.

The mediation path coefficient diagram is presented in Figure S3. More prominent CM were significantly predicted heightened DIF (β = .34, SE = .09, CI = [.69, .1.04]), DDF(β = .42, SE = .05, CI = [.53, .73]) and EOT(β = .26, SE = .06, CI = [.31, .55]). Heightened DIF significantly predicted elevated PD (β = .31, SE = .03, CI = [.15, .28]), whereas DDF (β = -.003, SE = .06, CI = [-.12, .12]) and EOT(β = -.001, SE = .04, CI = [-.08, .08]) were not significantly predicted elevated PD. The indirect effect showed that the DIF served as a significant mediator between CM and PD (β = .10, SE = .02, CI = [.06, .15]). The DDF and EOT did not significantly mediated this relationship.

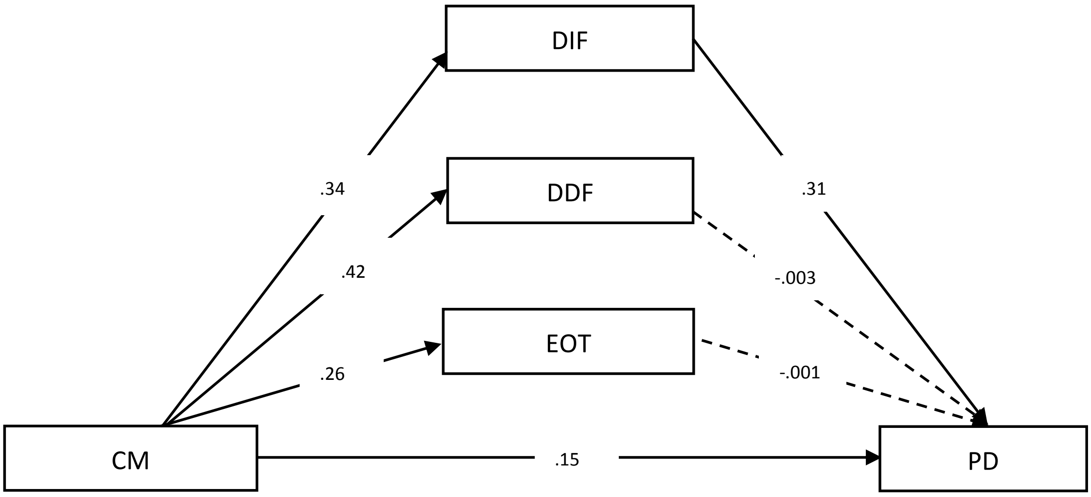

Figure S3 Mediation path coefficients with CM as the independent variable, DIF, DDF, and EOT as mediators, and PD as the dependent variable.

Results of the mediation analysis with IM as the independent variable, DIF, DDF, and EOT as mediators, and PD as the dependent variable.

The mediation path coefficient diagram is presented in Figure S4. More prominent IM were significantly predicted heightened DIF (β = .13, SE = .12, CI = [.19, .65]), DDF (β = .20, SE = .07, CI = [.24, .50]) and EOT (β = .17, SE = .07, CI = [.19, .48]). Heightened DIF significantly predicted elevated PD (β = .32, SE = .03, CI = [.16, .29]), whereas DDF (β = .06, SE = .06, CI = [-.15, .18]) and EOT (β = .03, SE = .04, CI = [-.05, .11]) were not significantly predicted elevated PD. The indirect effect showed that the DIF served as a significant mediator between IM and PD (β = .04, SE = .01, CI = [.02, .07]). The DDF and EOT did not significantly mediated this relationship.

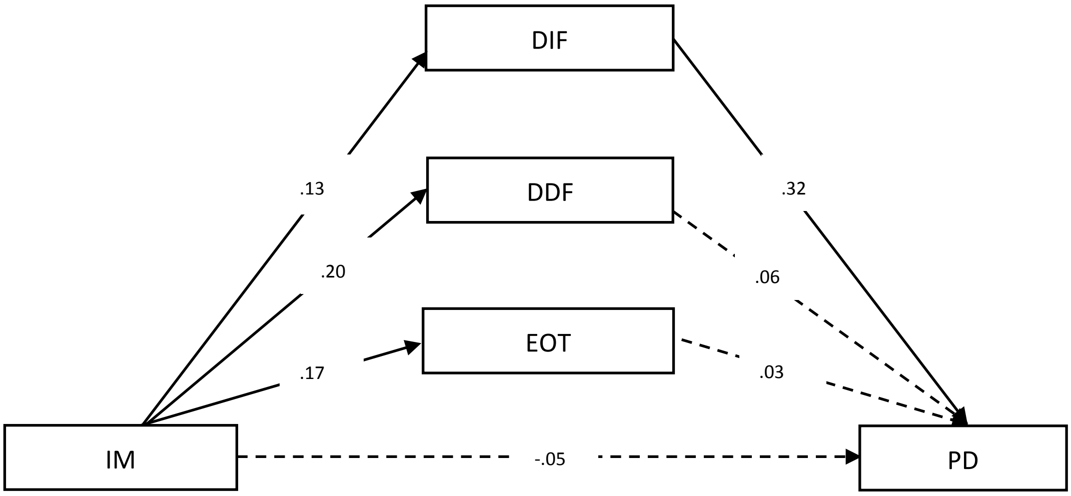

Figure S4 Mediation path coefficient diagram with IM as the independent variable, DIF, DDF, and EOT as mediators, and PD as the dependent variable.

Results of the mediation analysis with AD as the independent variable, DIF, DDF, and EOT as mediators, and PD as the dependent variable.

The mediation path coefficient diagram is presented in Figure S5. The AD were not significantly predicted DIF (β = .04, SE = .10, CI = [-.08, .30]) and DDF (β = -.07, SE = .06, CI = [-.22, .002]) but significantly predicted EOT (β = -.20, SE = .06, CI = [-.46, -.22]). Heightened DIF significantly predicted elevated PD (β = -.35, SE = .03, CI = [.17, .31]), whereas DDF (β = .03, SE = .06, CI = [-.08, .15]) and EOT(β =-.005, SE = .04, CI = [-.09, .07]) were not significantly predicted elevated PD. The indirect effects indicated that DIF, DDF, and EOT did not significantly mediate the relationship between AD and PD.

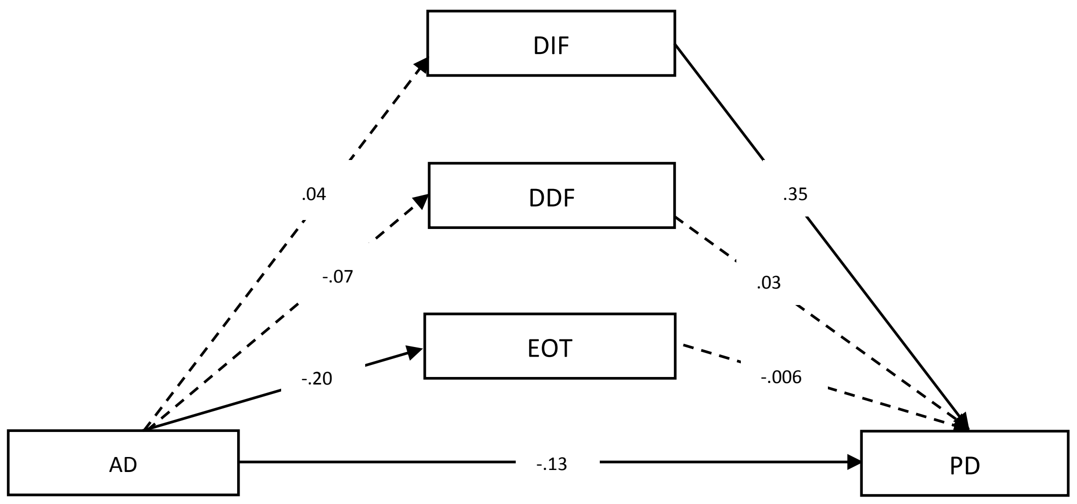

Figure S5 Mediation path coefficient diagram with AD as the independent variable, DIF, DDF, and EOT as mediators, and PD as the dependent variable.

Results of the mediation analysis with SS as the independent variable, DIF, DDF, and EOT as mediators, and EC as the dependent variable.

The mediation path coefficient diagram is presented in Figure S6. More prominent SS were significantly predicted heightened DIF (β = .21, SE = .07, CI = [.28, .57]), DDF (β = .29, SE = .04, CI = [.25, .41]) and EOT (β = .15, SE = .05, CI = [.09, .28]). The DIF (β = .10, SE = .04, CI = [-.003, .15]) and DDF (β = -.09, SE = .07, CI = [-.26, .01]) were not significantly predicted EC, whereas EOT(β =-.13, SE = .05, CI = [-.25, -.06]) was significantly predicted reduced EC. The indirect effect showed that the EOT served as a significant mediator between SS and EC (β =-.02, SE = .09, CI = [-.04, -.004]). The DIF and DDF did not significantly mediated this relationship.

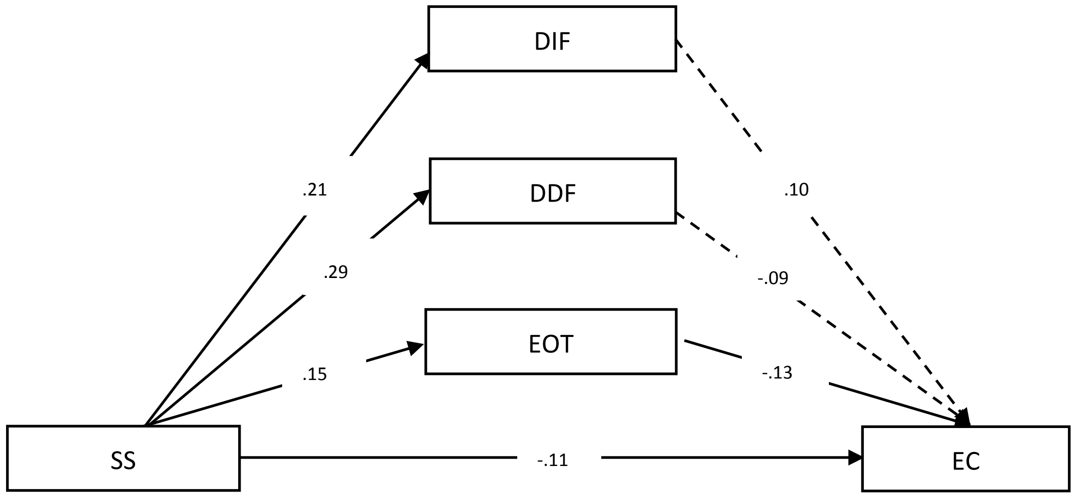

Figure S6 Mediation path coefficient diagram with SS as the independent variable, DIF, DDF, and EOT as mediators, and EC as the dependent variable.

Results of the mediation analysis with AS as the independent variable, DIF, DDF, and EOT as mediators, and EC as the dependent variable.

The mediation path coefficient diagram is presented in Figure S7. More prominent AS were significantly predicted heightened DIF (β = .26, SE = .10, CI = [.54, .94]), DDF (β = .22, SE = .06, CI = [.25, .48]) and EOT (β = .12, SE = .07, CI = [.09, .35]). The DIF (β = .09, SE = .04, CI = [-.008, .15]) was not significantly predicted EC, whereas the DDF (β = -.12, SE = .07, CI = [-.30, -.03]) and EOT(β =-.14, SE = .05, CI = [-.26, -.07]) were significantly predicted reduced EC. The indirect effect showed that the DDF (β = -.03, SE = .01, 95% CI = [-.06, -.002]) and EOT (β = -.02, SE = .01, CI = [-.03, -.004]) served as a significant mediator between AS and EC . The DIF did not significantly mediated this relationship.

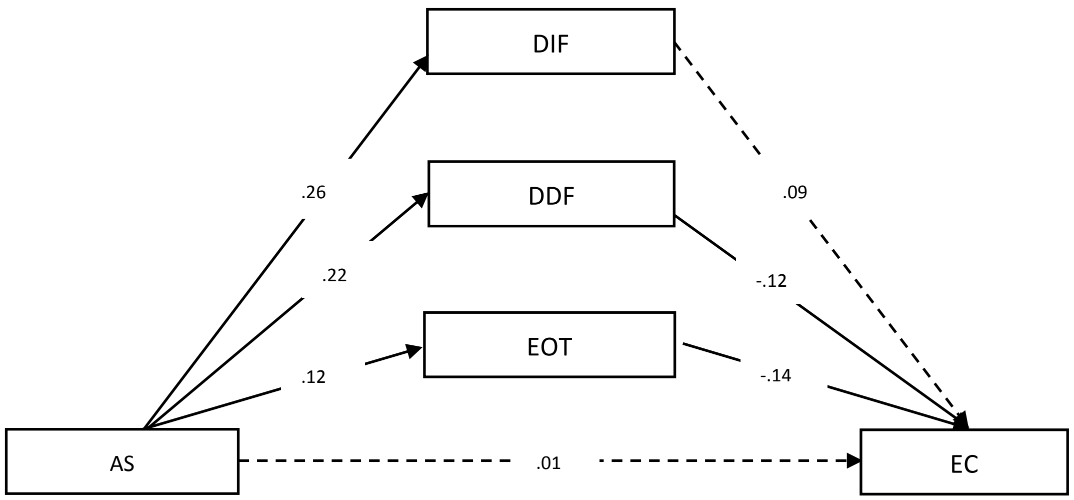

Figure S7 Mediation path coefficient diagram with AS as the independent variable, DIF, DDF, and EOT as mediators, and EC as the dependent variable.

Results of the mediation analysis with CM as the independent variable, DIF, DDF, and EOT as mediators, and EC as the dependent variable.

The mediation path coefficient diagram is presented in Figure S8. More prominent CM were significantly predicted heightened DIF (β = .34, SE = .09, CI = [.69, 1.04]), DDF (β = .42, SE = .05, CI = [.53, .73]) and EOT (β = .26, SE = .06, CI = [.32, .55]). The DIF (β = .10, SE = .04, CI = [.002, .16]) and EOT (β =-.12, SE = .05, CI = [-.23, -.05]) were significantly predicted elevated EC, whereas DDF (β = -.08, SE = .07, CI = [-.24, .04]) was not significantly predicted elevated EC. The indirect effect showed that the EOT (β = -.03, SE = .01, CI = [-.06, -.006]) served as a significant mediator between CM and EC . The DIF and DDF did not significantly mediated this relationship.

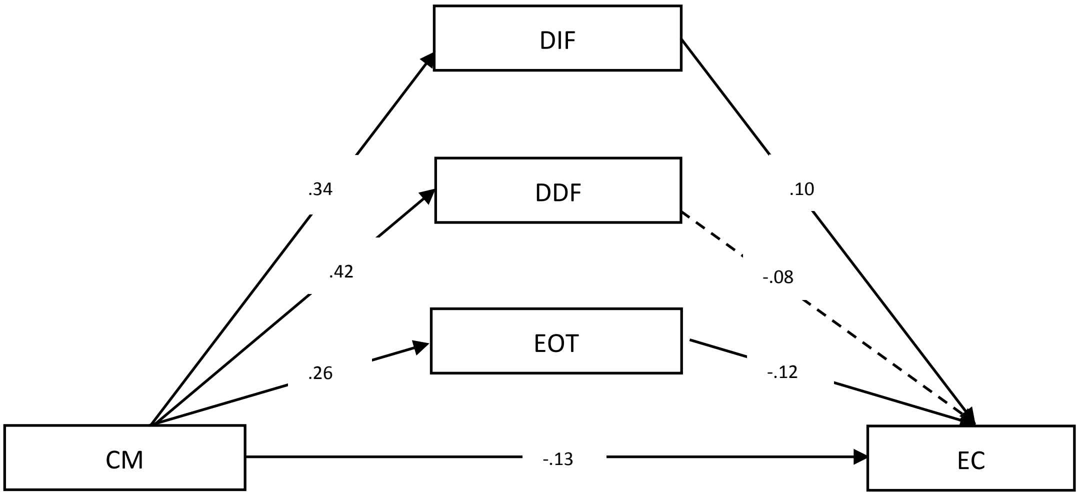

Figure S8 Mediation path coefficient diagram with CM as the independent variable, DIF, DDF, and EOT as mediators, and EC as the dependent variable.

Results of the mediation analysis with IM as the independent variable, DIF, DDF, and EOT as mediators, and EC as the dependent variable.

The mediation path coefficient diagram is presented in Figure S9. More prominent IM were significantly predicted heightened DIF (β = .13, SE = .12, CI = [.19, .65]), DDF (β = .20, SE = .07, CI = [.24, .50]) and EOT (β = .17, SE = .07, CI = [.19, .48]). The DIF (β = .09, SE = .04, CI = [-.007, .15]) and DDF (β = -.10, SE = .07, CI = [-.26, .01]) were not significantly predicted elevated EC, whereas the EOT(β =-.13, SE = .05, CI = [-.24, -.06]) was significantly predicted elevated EC. The indirect effect showed that the EOT (*β* = -.02, *SE* = .01, CI = [-.04, -.005]) served as a significant mediator between IM and EC . The DIF and DDF did not significantly mediated this relationship.

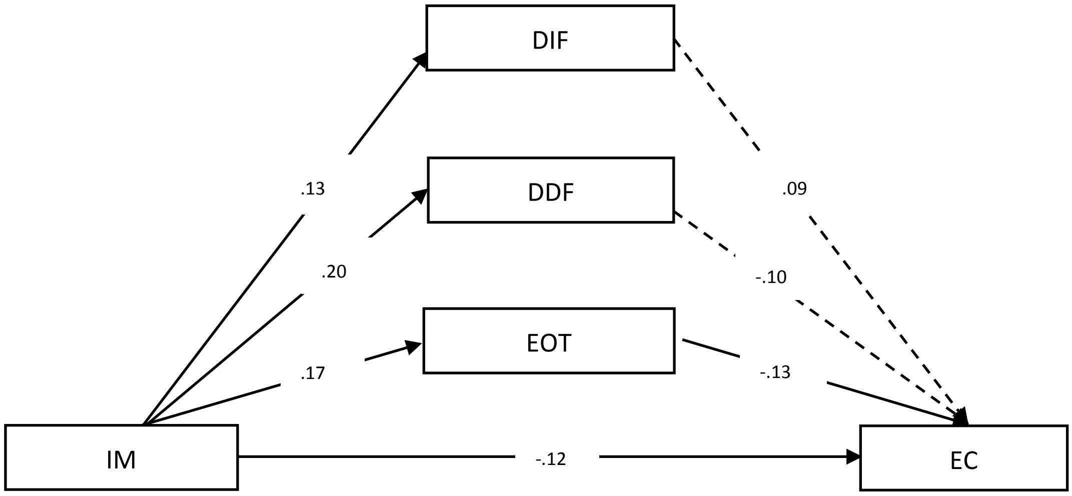

Figure S9 Mediation path coefficient diagram with IM as the independent variable, DIF, DDF, and EOT as mediators, and EC as the dependent variable.

Results of the mediation analysis with AD as the independent variable, DIF, DDF, and EOT as mediators, and EC as the dependent variable.

The mediation path coefficient diagram is presented in Figure S10. The AD were not significantly predicted DIF (β = .04, SE = .10, CI = [-.08, .30]), DDF (β = -.07, SE = .06, CI = [-.22, .002]) EC, but significantly predicted EOT (β = -.20, SE = .06, CI = [-.46, -.22]). The DIF (β = .08, SE = .04, CI = [-.02, .14]) was not significantly predicted elevated EC, whereas the DDF (β = -.11, SE = .07, CI = [-.28, -.01]) and EOT(β =-.13, SE = .05, CI = [-.25, -.06]) were significantly predicted EC. The indirect effect showed that the EOT served as a significant mediator between AD and EC (β = .02, SE = .01, CI = [.005, .05]). The DIF and DDF did not significantly mediated this relationship.

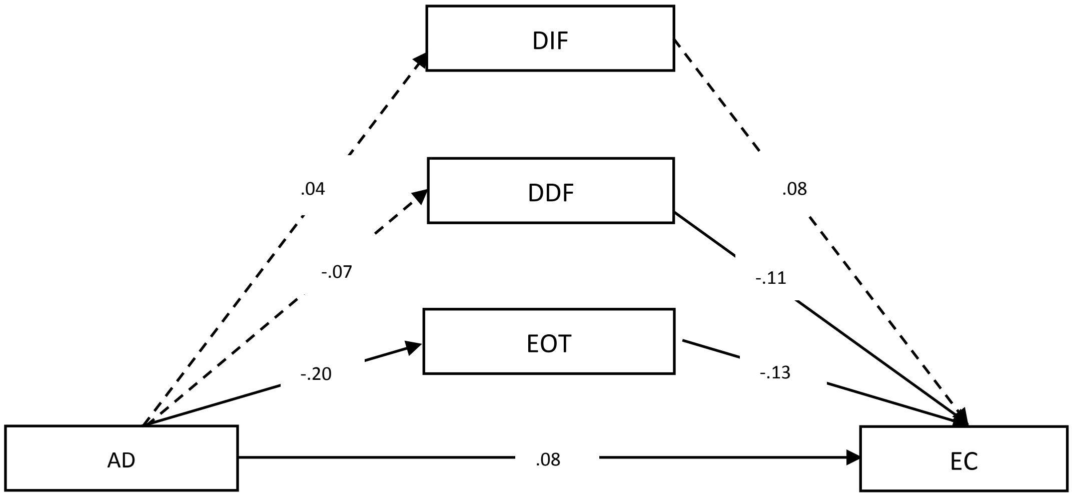

Figure S10 Mediation path coefficient diagram with AD as the independent variable, DIF, DDF, and EOT as mediators, and EC as the dependent variable.
